# Emergence of drug-resistant *Klebsiella pneumoniae* phylogroups (*K. quasipneumoniae* and *K. variicola*) causing human infections

**DOI:** 10.1128/spectrum.00198-25

**Published:** 2025-08-08

**Authors:** Kajal Mishra, Tuhina Banerjee, Ghanshyam Yadav, Ashok Kumar, Arvind Pratap, Sandhya Chaurasiya, Pue Rakshit

**Affiliations:** 1 Department of Microbiology, Institute of Medical Sciences, Banaras Hindu University 30114, Varanasi, Uttar Pradesh, India; 2 Department of Anaesthesiology, Institute of Medical Sciences, Banaras Hindu University 30114, Varanasi, Uttar Pradesh, India; 3 Department of Paediatrics, Institute of Medical Sciences, Banaras Hindu University 30114, Varanasi, Uttar Pradesh, India; 4 Department of General Surgery, Institute of Medical Sciences, Banaras Hindu University 30114, Varanasi, Uttar Pradesh, India; Duke University, Durham, North Carolina, USA

**Keywords:** phylogeny groups, colistin resistance, clinical, *PhoP*/*PhoQ*, *bla*_LEN_

## Abstract

This prospective, cross-sectional study was undertaken to identify the emerging *K. pneumoniae* phylogeny groups (KpI, KpII, KpIII) and characterize their drug resistance. Phylogeny groups of 150 clinical isolates of biochemically identified *K. pneumoniae* were detected by targeting their chromosomal class A, β-lactamase genes *bla*_SHV_, *bla*_LEN_, and *bla*_OKP_, respectively, and their flanking gene (*deoR*). Antimicrobial susceptibility testing was done by disk diffusion and broth microdilution methods and statistically analyzed. Carbapenemases (Classes A, B, and D) were detected by multiplex PCR. Colistin resistance mechanisms to detect alteration in *PhoP*/*PhoQ*, *pmrAB* two-component signaling pathways, and *mgrB* were done by PCR and sequencing. Of the total, KpI, *K. pneumoniae* were 93 (62%), KpII, *K. quasipneumoniae* were 36 (24%), and KpIII, *K. variicola* were 21 (14%). Carbapenem resistance was in 77 (51.3%); 52 (55.9%), 17 (47.2%), and 8 (38%) in KpI, KpII, and KpIII, respectively. Colistin resistance was in 16 (10.6%), 11 (68.75%) in KpI and 5 (31.25%) in KpIII. *K. variicola* was resistant to polymyxin B as compared with KpI (*P* = 0.0008). *bla*_NDM_ (63, 81.8%) was the commonest. Co-harboring of multiple carbapenemase genes was significant in all the phylogroups (*P* < 0.001). The majority of the cases of *K. variicola* were males (*P* = 0.0139) and in the intensive care unit (*P* = 0.0091). Several non-synonymous mutations were observed in the colistin-resistant isolates in *PhoP* and *PhoQ* genes, with the phylogenetic tree revealing different evolutionary relationships among the isolates. There is considerable emergence of *K. quasipneumoniae* and *K. variicola* as prominent human pathogens along with drug resistance, which requires attention.

**IMPORTANCE:**

These epidemiological data add to the extremely scarce literature on the occurrence of the two phylogeny groups of *K. pneumoniae*, namely *K. quasipneumoniae* and *K. variicola*, in infections and highlight their widespread dissemination as prominent human pathogens, beyond agriculture and environment as their common habitat. There was significant drug resistance in the phylogeny groups, including colistin resistance in *K. variicola*, which was studied for the first time.

## INTRODUCTION

The World Health Organization (WHO) has declared antimicrobial resistance (AMR) as one of the top 10 most serious global public health threats facing humanity (1). In order to emphasize the urgency for the development of newer antimicrobials to combat the drug-resistant pathogens, it has prioritized bacterial pathogens into categories of critical, high, and medium priority (2). In this respect, carbapenem-resistant *Klebsiella pneumoniae* (CRKP) has been prioritized as a “critical pathogen” of utmost priority, both by WHO and in the Indian context (3, 4). CRKP has been reported in over 50 countries, with a high rate of resistance in Asia, Europe, and the Americas (5). Based on global data, CRKP has been linked to high mortality rates, with case fatality rates ranging from 20% to 50% (6). Besides, analysis of AMR Surveillance data for the year 2020 has revealed that *Klebsiella* species is the second most common pathogen isolated among outpatients and inpatients (7).

The description of the emerging species has expanded the taxonomic classification of the genus *Klebsiella*, which is described as *K. pneumoniae* complex. The *K. pneumoniae* complex consists of seven members, including *K. pneumoniae* subsp. *pneumoniae* (KpI), *K. quasipneumoniae* subsp. *quasipneumoniae* (KpII), *K. quasipneumoniae* subsp. *similipneumoniae,* and *K. variicola* subsp. *variicola* (KpIII) (8). All these species are biochemically and phenotypically identical, thus showing overlapping properties. Phylogenetic analysis has helped in distinguishing between closely related species within the complex, ensuring accurate identification and diagnosis (9). It is worth mentioning that *K. variicola* has been considered an emerging pathogen in humans. However, accurate differentiation of *K. pneumoniae*, *K. quasipneumoniae*, and *K. variicola* is not routinely performed in a clinical setting. All these species in the complex are misidentified as *K. pneumoniae*. AMR is widely distributed in several species of *K. pneumoniae* complex, but there is a lack of epidemiological data in these aspects. Therefore, this study was planned to identify the *K. pneumoniae* phylogeny groups (KpI, KpII, KpIII) and characterize their drug resistance profile and probable mechanisms of drug resistance.

## MATERIALS AND METHODS

### Study site

This prospective, cross-sectional study was conducted in the Department of Microbiology, Institute of Medical Science, Banaras Hindu University, and the associated 2,500-bed tertiary care hospital.

### Bacterial strains

The study included 150 isolates of *K. pneumoniae* collected from various clinical samples over a period of 1 year. For this, clinical samples, namely blood, urine, aspirated pus, endotracheal aspirates, and other body fluids from patients admitted to intensive care units (ICUs), neonatal ICUs (NICUs), and wards, with suspected clinical infection were inoculated onto blood agar and MacConkey agar. Isolates of *K. pneumoniae* were presumptively identified by the standard biochemical tests (10). Only those isolates were selected for the study in which, besides the above criteria of being clinically relevant, required demographical data were available. Those isolates with incomplete data and for which consent was denied were excluded for the study.

### Antibiotic susceptibility testing

The *in vitro* susceptibility pattern of isolates was tested against the following standard antibiotics: amoxicillin (30 µg), piperacillin/tazobactam (100/10 µg), ampicillin/sulbactam (10/10 µg), ceftriaxone (30 µg), cefuroxime (30 µg), cefazolin (30 µg), meropenem (10 µg), imipenem (10 µg), amikacin (30 µg), gentamicin (10 µg), ciprofloxacin (5 µg), levofloxacin (5 µg), nitrofurantoin (30 µg) for urinary isolates, and trimethoprim/sulfamethoxazole (1.24/23.75 µg) (HiMedia Laboratories Pvt. Ltd., India) by Kirby Bauer disk diffusion method. Additionally, susceptibility to polymyxin B, aztreonam, ceftazidime (Sigma-Aldrich Chemicals Pvt. Ltd., India), and avibactam (MedChem Express, USA) was done by broth microdilution method as per standards. Colistin susceptibility testing was done by colistin broth disk elution method (CBDE) (Becton, Dickinson and Company, India) as per Clinical and Laboratory Standards Institute (CLSI) standards (11). Screening for carbapenem resistance was done by broth microdilution method against meropenem and doripenem (Sigma- Aldrich Chemicals Pvt. Ltd., India). Carbapenem resistance was defined as resistance to one or several carbapenem antibiotics (12). Isolates were classified as multidrug resistant (MDR) and extensively drug resistant (XDR) as per reference (13). Standard strains *Escherichia coli* ATCC 25922 and *Pseudomonas aeruginosa* ATCC 27853 were used as quality control strains (14).

### Genotyping of isolates to differentiate *K. pneumoniae* phylogeny groups

All the biochemically confirmed isolates were subjected to semi-multiplex PCR to detect different phylogeny groups as *K. pneumoniae* (KpI), *K. quasipneumoniae* (KpII), and *K. variicola* (KpIII) by targeting their chromosomal class A, β lactamase genes *bla*_SHV_, *bla*_OKP_, and *bla*_LEN_, respectively, and their flanking gene (*deoR*). PCR was performed under the following amplification condition: an initial denaturation at 95°C for 5 min followed by 40 amplification cycles. Denaturation at 94°C for 30 s, annealing at 55°C for 30 s, extension at 72°C for 1 min, final extension at 72°C for 10 min. The amplicons were analyzed on 2% agarose gel electrophoresis at 60 V for 2 h (15). *Klebsiella* ATCC 700603 (*K. quasipneumoniae*) and *K. variicola* ATCC 31488 were used as internal controls for validation of the PCR.

### Detection of class A, B, and D carbapenemase genes

All carbapenem-resistant isolates were screened for the presence of class A (*bla*_SME_, *bla*_NMC_, *bla*_GES_, *bla*_KPC_), class B (*bla*_IMP_, *bla*_VIM_, *bla*_NDM_), and class D (*bla*_OXA_-_48_) carbapenemases by multiplex and conventional PCR, using primers and reaction conditions described elsewhere (16–18).

### Extraction of plasmid DNA and characterization of extended spectrum β-lactamases (ESBLs), and class 1 and 2 integrons

Plasmid DNA extraction was carried out using QIAprep Spin Miniprep kit (Qiagen, Germany) as per the manufacturer’s instructions. Multiplex PCR from the plasmid DNA was performed to detect the ESBL genes utilizing primers for *bla*_TEM_, *bla*_SHV_, *bla*_CTXM_ as described elsewhere (19). All the colistin-resistant isolates were screened for the presence of the above-mentioned carbapenemases as well as screened for the presence of integrons through PCR for integrase class 1 and 2 genes (20).

### Characterization of colistin resistance mechanisms

Conventional PCR was performed to detect alteration in *PhoP*/*PhoQ*, *pmrAB*, two-component signaling pathways, and *mgrB* (21). Following amplifications, the positive amplicons were purified (QIAquick purification kit, Qiagen, Germany) for Sanger sequencing (Barcode Biosciences, India). The sequencing results were analyzed using Basic Local Alignment Search Tool (BLAST) http://www.ncbi.nlm.nih.gov/BLAST and compared with reference genome *K. pneumoniae* ATCC 700603. Mutations in genes involved in resistance were detected using ClustalW multiple sequence alignment tool. Following this, in vitro nucleotide translation was performed on the ExPASY translate tool. These translated sequences were again aligned in ClustalW multiple sequence alignment tool, and changes in amino acid sequences were noted. The functional impacts of the mutations were assessed by using the Sorting Intolerant From Tolerant (SIFT) tool (https://bio.tools/t?operationID=%22operation_1777%22). The phylogenetic tree was constructed using the neighbor-joining method in the ClustalW tool of MEGA software (version 11.0.13). Branch length was computed using the maximum likelihood method and is present beside the branches.

For the detection of mobile colistin resistance determinants (*mcr-1, mcr-2, mcr-3, mcr-4, mcr-5*), multiplex PCR was performed as per the condition and primer described elsewhere (22).

### Statistical analysis

Demographic factors (age, male gender, ICU admission) were compared by χ test among the phylogroups. The rate of drug resistance among different phylogeny groups and the distribution of carbapenemase encoding genes was compared among different phylogeny groups by χ test using MedCalc software (version 22.032).

## RESULTS

### Bacterial isolates and phylogeny groups

**Fig 1:**
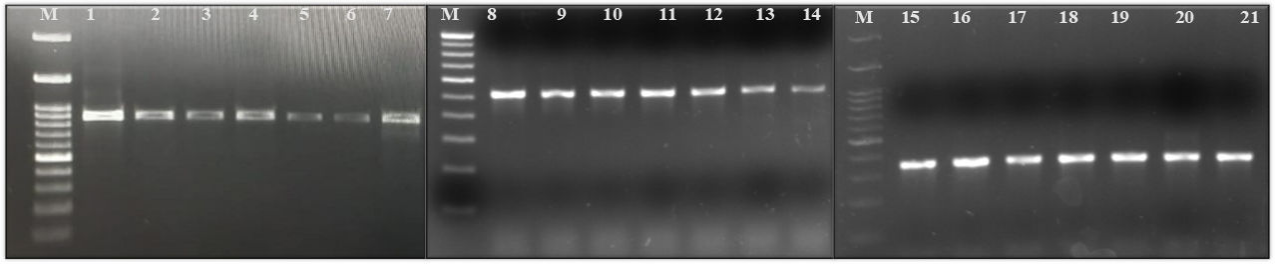
Gel image of multiplex PCR for identification of *Klebsiella pneumoniae* phylogeny group. Lane M is 100 bp molecular marker. Lanes 1–7 are *bla*_SHV_ at 995 bp (*Klebsiella pneumoniae*). Lanes 8–14 are* bla*_LEN_ at 485 bp (*Klebsiella variicola*). Lanes 15–21 are *bla*_OKP_ at 348 bp (*Klebsiella quasipneumoniae*).

A total of 150 clinical isolates of *K. pneumoniae* were studied, of which the majority 66 (44%) were from urine, 50 (33.3%) from pus, and 34 (22.6%) from blood. Genotyping of all these isolates revealed that the majority belonged to the phylogeny group KpI, *K. pneumoniae* (93, 62%), distributed over 41 (44%) urine samples, 32 (34.4%) pus samples, and 20 (21.5%) blood samples. Phylogeny group KpII, *K. quasipneumoniae* was the second most common isolate (36, 24%), distributed over 14 (38.8%), 13 (36.1%), and 9 (25%) urine, pus, and blood samples, respectively. The third common group was KpIII, *K. variicola* (21, 14%), which was predominant in 11 (52.3%) isolates in urine, followed by 5 (23.8%) and 5 (23.8%) isolates in blood and pus samples, respectively. The PCR amplified representation of the three groups has been shown in Fig. 1.

The demographic profile of the patients revealed that the mean ages of the study groups were comparable, 41.75 ± 18.31, 39.54 ± 25.75, and 43.18 ± 18.65 years for KpI, KpII, and KpIII, respectively. The proportion of male patients was 52.68% (49/93) in KpI, 66.66% (24/36) in KpII, and significantly more in 85.71% (18/21) in KpIII (*P* = 0.0139). Among the study population, ICU admission was recorded in 18.75% (18/96) of KpI cases, 36.11% (13/36) of KpII, and significantly more in 47.61% (10/21) of KpIII cases (*P* = 0.0091).

### Antimicrobial susceptibility testing

The disk diffusion data revealed non-susceptibility against the majority of the antibiotics as shown in Table 1. Among the 150 isolates, 26.66% (40 of 150) were MDR and 4% (6 of 150) were XDR, with the majority of the MDR isolates being KpIII followed by KpII and KpI. A statistically significant difference in nitrofurantoin susceptibility was noted among the KpI urinary isolates as compared with the other phylogroup (*P* = 0.04).

**TABLE 1:** Drug resistance profile of the studied isolates based on disk diffusion assay

Antimicrobial category	Antimicrobials	No. of resistant isolates, *N* (%)			Total *N* (%)	*P* value
		*Klebsiella pneumoniae* (KpI, 93)	*Klebsiella quasipneumoniae* (KpII, 36)	*Klebsiella variicola* (KpIII, 21)		
1st generation cephalosporins	Cephazolin	64 (68.81)	31 (86.11)	14 (66.66)	109 (72.66)	0.11
2nd/3rd generation cephalosporins	Cefuroxime	52 (55.91)	27 (75.00)	13 (61.90)	92 (61.33)	0.09
	Cefotaxime	48 (51.61)	26 (72.22)	12 (57.14)	86 (57.33)	0.10
Penicillin+β-lactamase inhibitors	Piperacillin- tazobactam	67 (72.04)	31 (86.11)	17 (80.95)	115 (76.66)	0.20
	Amoxicillin - clavulanate	65 (69.89)	27 (75.00)	17 (80.95)	109 (72.66)	0.55
Aminoglycosides	Gentamicin	52 (55.91)	26 (72.22)	13 (61.90)	91 (60.66)	0.23
	Amikacin	52 (55.91)	25 (69.44)	13 (61.90)	90 (60.00)	0.36
Fluoroquinolones	Ciprofloxacin	52 (55.91)	26 (72.22)	13 (61.90)	91 (60.66)	0.23
	Levofloxacin	52 (55.91)	26 (72.22)	13 (61.90)	91 (60.66)	0.23
Folate pathway inhibitors	Trimethoprim-sulfamethoxazole	51 (54.83)	26 (72.22)	13 (61.90)	90 (60.00)	0.23
Nitrofurans^*a*^	Nitrofurantoin	17 (50)	8 (23.52)	9 (26.47)	34 (51.51)	0.04^*a*^
Carbapenems	Imipenem	52 (55.91)	17 (47.22)	8 (38.09)	77 (57.33)	0.28
	Doripenem	44 (47.31)	19 (52.77)	12 (57.14)	75 (50.00)	0.66
MDR/XDR strains		MDR= 19 (20.43) XDR = 3 (3.22)	MDR= 13 (36.11) XDR = 2 (5.55)	MDR = 8 (38.09) XDR = 1 (04.76)	MDR= 40 (26.66) XDR = 6 (4)	

^*a*^ Urine isolates only; MDR = multi-drug resistant; XDR = extensively drug resistant.

By MIC determination, as shown in Table 2, carbapenem resistance was detected in 77 (51.3%) isolates, comprising of 52 (55.9%), 17 (47.2%), and 8 (38%) in KpI, KpII, and KpIII, respectively. Colistin resistance was found in 16 (10.6%) isolates, 11 (68.75%) in KpI and 5 (31.25%) in KpIII. Polymyxin B resistance was found in 28 isolates (18.66%), of which *K. variicola* isolates were significantly resistant as compared with KpI (*P* = 0.0008). A total of 45 (30.00%) isolates were resistant to ceftazidime-avibactam, the majority being in KpI phylogeny group.

**TABLE 2:** Summary of antibiotic resistance pattern in phylogeny group of *K. pneumoniae* based on broth microdilution method

Antibiotics	Total (*N* = 150)		KpI = 93		KpII = 36		KpIII = 21	
	*n*	%	*n*	%	*n*	%	*n*	%
Meropenem	77	51.33	52	55.91	17	47.22	8	38.09
Doripenem	75	50.00	44	47.32	19	52.77	12	57.15
Ceftazidime/avibactam	45	30.00	29	31.18	10	27.77	6	28.57
Polymyxin B^*a*^	28	18.66	11	11.82	8	22.22	9	42.85^*a*^
Colistin	16	10.66	11	13.97	0	00.00	5	14.28

^*a*^ *P* = 0.0008.

### Carbapenemase-encoding genes

Class B carbapenemases were seen in varying proportions with *bla*_NDM_, 63 (81.8%) being the commonest in all the CRKP isolates as shown in Table 3. This gene, *bla*_NDM_, was predominant in phylogeny group KpI in 51 (80.95%) isolates, followed by 6 (9.52%), 6 (9.52%) in phylogeny groups Kp II and Kp III, respectively. Among the class D carbapenemases, *bla*_OXA-48_-positive isolates were 47 (61%), predominantly seen in phylogeny group KpI in 38 (80.85%), followed by 4 (8.51%) isolates in phylogeny group KpII and 5 (10.63%) isolates in KpIII group. The combination of *bla*_OXA-48_ with *bla*_NDM_ was found in 47 (61%) isolates (KpI, 38, 80.85%; KpII in 4, 8.51% isolates and Kp III in 5, 10.63% isolates). Co-harboring of multiple carbapenemase genes in all the phylogroups KpI, KpII, and KpIII was found to be statistically significant (*P* < 0.0001).

**TABLE 3:** Distribution of carbapenemase-encoding genes among the *K. pneumoniae* phylogroups^*a*^

Bacterial isolates	Carbapenemase genes (*n* = 77)						
	*bla*_NDM_ (*n* = 63)	*bla*_VIM_ (*n* = 39)	*bla*_IMP_ (*n* = 31)	*bla*_OXA-48_ (*n* = 47)	*bla*_NDM_ + *bla*_OXA-__48_ (*n* = 47)	*bla*_NDM_ + *bla*_VIM_ (*n* = 28)	*bla*_NDM_ + *bla*_VIM_ + *bla*_OXA-48_ (*n* = 19)
CRKpI (52)	51 (80.95)	31 (79.48)	24 (77.41)	38 (80.85)	38 (80.85)	21 (74.99)	14 (73.68)
CRKpII (17)	6 (9.52)	5 (12.82)	5 (16.12)	4 (8.51)	4 (8.51)	4 (14.28)	3 (15.78)
CRKpIII (8)	6 (9.52)	3 (7.69)	2 (6.45)	5 (10.63)	5 (10.63)	3 (10.71)	2 (10.52)

^*a*^ *P* < 0.0001 for isolates carrying more than one carbapenemase genes in all the three groups.

None of the isolates showed the presence of β-lactamase genes in their plasmids except one, which harbored *bla*_NDM_ gene in its plasmid. Class 1 integron was found in 4 of the 5 (80%) colistin-resistant isolates of KpI but in none of the resistant isolates of KpIII.

### Study of the mechanism of polymyxin resistance

Several mutations were observed in the colistin-resistant isolates in the studied genes of *PhoP* and *PhoQ* two-component regulatory system. Four isolates of KpI, *K. pneumoniae* (1, 2, 3, 5) and four isolates of KpIII *K. variicola* (1, 2, 3, 4) showed nucleotide deletion for *PhoP* gene as shown in Table 4. Five isolates of KpI (1, 2, 3, 4, 5) and four isolates of KpIII (2, 3, 4, 5) showed nucleotide deletion in the *PhoQ* gene. All the mutations were predicted to be non-synonymous mutations by the SIFT predictive tool. None of the isolates showed the presence of *mcr* genes.

**TABLE 4:** Profile of colistin-resistant isolates of KpI and KpIII phylogroups^*a*^

Isolate no.	Carbapenemase genes			ESBL genes	*mcr* genes	Nucleotide change		Integrons
	Class A	Class B	Class D		–	PhoP	PhoQ	
KpI-1	**–**	*bla*_IMP_	**–**	*bla*_SHV_	**–**	Deletion at M, 262	Deletion at W, 141	Class 1
KpI-2	**–**	*bla*_NDM_	*bla*_OXA-48_	*bla*_SHV_ + *bla*_CTXM_	**–**	Deletion at E, 23	Deletion at G, 115	Class 1
KpI-3	**–**	*bla*_NDM_ + *bla*_VIM_	*bla*_OXA-48_	*bla*_SHV_	**–**	Deletion at G, 69	Deletion at L, 209	Class 1
KpI-4	**–**	*bla*_NDM_ + *bla*_VIM_	**–**	*bla*_SHV_ + *bla*_CTXM_	**–**	**–**	Deletion at P, 111	Class 1
KpI-5	**–**	*bla*_NDM_ + *bla*_VIM_	*bla*_OXA-48_	*bla*_SHV_ + *bla*_CTXM_	**–**	Deletion at E, 30	Deletion at K, 117	–
KpIII-1	**–**	*bla*_NDM_	*bla*_OXA-48_	*bla*_SHV_ + *bla*_CTXM_	**–**	Deletion at K, 334	**–**	–
KpIII-2	**–**	*bla*_IMP_		*bla*_CTXM_ + *bla*_SHV_	**–**	Deletion at Y, 65	Deletion at Y, 65	–
KpIII-3	**–**	*bla*_NDM_	*bla*_OXA-48_	*bla*_CTXM_ + *bla*_SHV_	**–**	Deletion at C, 79	Deletion at C, 79	–
KpIII-4	**–**	*bla*_NDM_ + *bla*_VIM_	**–**	**–**	**–**	Deletion at P, 111	Deletion at I, 129	–
KpIII-5	**–**	*bla*_NDM_ + *bla*_VIM_	*bla*_OXA-48_	**–**	**–**	**–**	Deletion at K, 120	–

^*a*^ –, not present.

**Fig 2:**
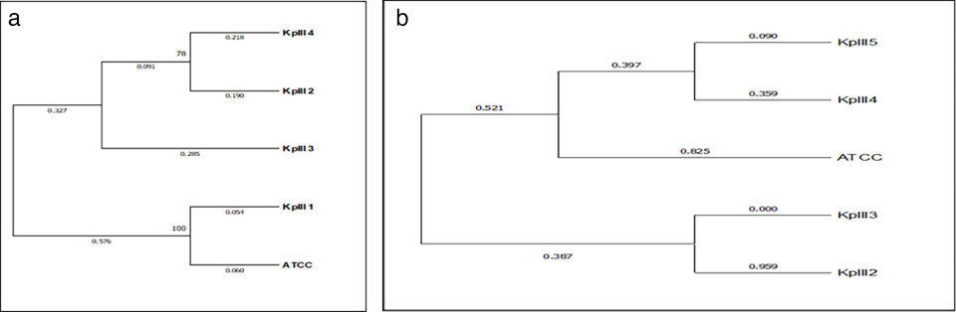
(a) Phylogenetic tree for PhoP gene. The phylogenetic tree for *PhoP* gene was generated using the neighbor-joining (distance-based) method. Bootstrap confidence estimates were generated by running 1,000 trials to determine the support for each branch in the phylogenetic tree. The evolutionary distances in terms of base substitutions per site were determined using the maximum composite likelihood approach. (b) Phylogenetic tree for PhoQ gene. The phylogenetic tree for PhoQ gene was constructed using neighbor-joining (distance-based) method. Each branch in the phylogenetic tree was supported by 1,000 bootstrap confidence values. The maximum composite likelihood technique was used to calculate the evolutionary distances in terms of base substitutions per site.

Phylogenetic tree for *PhoP* and *PhoQ* gene revealed that both the trees were different in terms of evolutionary relationship among the isolates. Based on the *PhoP* gene, Fig. 2a reveals a close relation of KpIII isolate one with the reference ATCC strain, while KpIII 2, 3, and 4 had different clades. Based on the *PhoQ* gene, the phylogenetic tree was divided into two clades from a single node. Among all the isolates, KpIII isolates 4 and 5 were closely related to the reference ATCC strain, while isolates 2 and 3 belonged to a separate clade as shown in Fig. 2b.

## DISCUSSION

This study revealed the isolation of different phylogeny groups of *K. pneumoniae* from clinical specimens, which are otherwise misidentified as *K. pneumoniae*, and studied their antimicrobial microbial resistance pattern, genetic profile, and the antibiotic resistance mechanisms. There has been extreme scarcity of data on the frequency of isolation and antibiotic resistance in these groups in literature (8). Though the majority of isolates were *K. pneumoniae* (KpI), as also reported in previous studies, there was a notable detection rate of *K. quasipneumoniae* and *K. variicola* from urine, pus, and blood samples of patients with clinical infections.

It has been noted that *K. pneumoniae,* along with *K. variicola,* is usually implicated in the majority of the urinary tract, respiratory tract, and bloodstream infections (BSI) (8). This study found a total of 14% *K*. *variicola* isolates, with 16.7% from urine, which was higher than that reported (6.2%) (23). Similarly, from blood, the isolation of *K. variicola* (14.7%) was higher and lower to those reported in previous studies (3.25% and 29.9%, respectively) (23, 24). Interestingly, the majority of the isolates of *K. variicola* were from male patients admitted in the ICU (*P* < 0.05). It has been noted that BSI due to *K. variicola* causes higher mortality than the other two related species (25). In this study, though all the *K. variicola* isolates from BSI were from ICU patients causing 100% mortality, we could not compare the outcome of the infections with the other phylogroups owing to unavailability of data. However, there have been studies that have evaluated the clinical characteristics of BSI due to the three species and found that there was no significant difference in the outcome of BSI, though all the species had differences in their pathogenicity and possession of hypervirulent genes (26). Comparable isolation rates have been reported in previous studies (2.1% and 2%, respectively) (27, 28). These data clearly document the emergence of *K. variicola* as a prominent pathogen in humans.

In a similar context, *K. quasipneumoniae* is a relatively new species that is related to both *K. pneumoniae* and *K. variicola*. However, hospital transmission, persistence, and survival mechanisms of *bla*_KPC_ positive *K. quasipneumoniae* have been described recently (29), thus emphasizing the importance of this emerging human pathogen. Otherwise, both *K. quasipneumoniae* and *K. variicola* were thought to be confined to agriculture and environment as their common habitat (29, 30). This study revealed a 24% occurrence of *K. quasipneumoniae* among the studied isolates with nearly 25% causing BSI. This rate could not be compared owing to the fact that there are scarce data on occurrence of *K. quasipneumoniae* in infections, its prevalence, ecological niche, and AMR (31).

This study revealed alarming resistance rates among the *K. variicola* and *K. quasipneumoniae* isolates, which are otherwise susceptible to antibiotics, highlighting the need for effective antimicrobial stewardship. Of the available data on AMR in *K. variicola*, the study reported higher resistance to carbapenems, ceftazidime-avibactam, and polymyxin B (26, 27, 31). However, these global studies emphasize the clinical outcome of *K. variicola* infections and do not throw much light upon the drug resistance pattern of the organism. There was a difference in the rates of resistance to colistin and polymyxin B as shown by all the isolates in the different phylogroups. Though both antimicrobial agents share a common resistance mechanism in targeting the lipid A modifications of the cell wall, the difference could be most likely explained in line with the fact that the interactions of colistin with lipid A are different from those of polymyxin B, which in turn could also target other cellular components (32). It was interesting to note that polymyxin B resistance was significantly higher in *K. variicola* (42.85%) as compared with other species. In addition, colistin resistance was seen in *K. variicola* and not in *K. quasipneumoniae* among these emerging species, the mechanism of which was studied in detail. Several mutations were noted in the two-component regulatory system *PhoP* and *PhoQ* genes. All the mutations were different from one another, and no pattern could be ascertained. These mutations were predicted to be non-synonymous, thus implying the potential ability of bringing about functional changes in the proteins by these mutations.

There was no substantial variation in the distribution of dominant type of carbapenemases in these isolates with *bla*_NDM_, predominance seen in *K. pneumoniae* isolates followed by *K. quasipneumoniae* and *K. variicola*. However, appreciable rates of co-occurrence of different carbapenemase-encoding genes, including *bla*_OXA-48_ along *bla*_NDM_, was seen in all the phylogroups, which were significant compared with harboring single carbapenemase genes. This makes these isolates unique and more challenging for management, even though nearly all these genes in the colistin-resistant isolates were not carried in their plasmid. As compared with previous studies, the occurrence of these carbapenemases was higher (23–25, 28), thus implying the emergence of these pathogens as drug-resistant organisms.

Finally, the study was not without limitation. As only 150 isolates were studied, an extensive data set on these phylogeny groups could have better elucidated the emerging issue of these isolates. Polymyxin B resistance could not be studied for all the resistant isolates against all the probable mechanisms. Nonetheless, the major strength of the study is that it gives the first-hand information of distribution of clinically relevant phylogeny groups of *K. pneumoniae* and their emerging drug resistance pattern. Further studies are needed to understand the epidemiology of clinically relevant *K. variicola* and *K. quasipneumoniae* in India. To the best of our knowledge, this is one of the first studies characterizing drug resistance and their mechanisms in the two emerging phylogeny groups of *K. pneumoniae*, namely *K. variicola* and *K. quasipneumoniae*.

### Conclusion

The study revealed 24% and 14% emergence of the two phylogeny groups of *K. pneumoniae*, namely *K. quasipneumoniae* and *K. variicola,* respectively, among the biochemically identified clinical isolates *K. pneumoniae* causing infections. There was significant drug resistance in the phylogeny groups, including colistin resistance in *K. variicola*. The majority of the cases with *K. variicola* infection were males (*P* = 0.0019) and from the ICU (*P* = 0.0139). These epidemiological data add to the extremely scarce literature on the occurrence of these two species in infections and highlight their widespread dissemination as prominent human pathogens, beyond agriculture and environment as their common habitat.

## References

[1] World Health Organization. 2020. Antimicrobial resistance: Klebsiella Pneumoniae. Available from: https://www.who.int/publications/i/item/9789240082496. Retrieved 12 Nov 2024.

[2] World Health Organization. 2024. Global priority list of antibiotic-resistant bacteria to guide research, discovery, and development of new antibiotics. Available from: https://www.who.int/news/item/17-05-2024

[3] World Health Organization 2021. New indian priority pathogen test to guide discovery of effective antibiotics. Available from: https://dbtindia.gov.in/ latest-announcement/indian-priority-pathogen-list-jointly-developed-dbt-and-who-india-office

[4] Centers for Disease Control and Prevention. 2020. Antibiotic resistance threats in the United States: Klebsiella Pneumoniae. Available from: https://www.cdc.gov/antimicrobial-resistance/data-research/threats/index.html

[5] Lan P, Jiang Y, Zhou J, Yu Y. 2021. A global perspective on the convergence of hypervirulence and carbapenem resistance in Klebsiella pneumoniae. J Glob Antimicrob Resist 25:26–34. doi:10.1016/j.jgar.2021.02.020 33667703

[6] National Antimicrobial Resistance Monitoring System for Enteric Bacteria (NARMS). 2020. Annual report*.* Available from: https://www.cdc.gov/ narms/reports/healthy-people-2020.html

[7] Li D, Huang X, Rao H, Yu H, Long S, Li Y, Zhang J. 2023. Klebsiella pneumoniae bacteremia mortality: a systematic review and meta-analysis. Front Cell Infect Microbiol 13:1157010. doi:10.3389/fcimb.2023.1157010 37153146 PMC10159367

[8] Rodríguez-Medina N, Barrios-Camacho H, Duran-Bedolla J, Garza-Ramos U. 2019. Klebsiella variicola: an emerging pathogen in humans. Emerg Microbes Infect 8:973–988. doi:10.1080/22221751.2019.1634981 31259664 PMC6609320

[9] Brisse S, Passet V, Grimont PA. 2013. Klebsiella variicola, a new species from human and animal sources. Syst Appl Microbiol 36:339–346. 10.1099/ijs.0.062737-0.23706914

[10] Crichton PB. 1996. “Enterobacteriaceae*: Escherichia, Klebsiella, Proteus* and other genera” in Mackie and McCartney practical medical microbiology, p 361–364. In Collee EdsJG, Mackie TJ, McCartney JE (ed), 14th ed. London, UK.

[11] Clinical Laboratory Standard Institute. 2023. Tests for colistin resistance in Enterobactarales. In Performance standard for antimicrobial susceptibility, M07-A13, 33rd ed. Clinical Laboratory Standard Institute, Wayne, PA.

[12] Clinical Laboratory Standard Institute. 2023. Introduction to tables 3B and 3C page no. 152.Test for carbapenems in *Enterobacterales*. In Performance standard for antimicrobial susceptibility, M07-A13, 33rd ed. Clinical and Laboratory Standards Institute, Wayne, PA.

[13] Magiorakos AP, Srinivasan A, Carey RB, Carmeli Y, Falagas ME, Giske CG, Harbarth S, Hindler JF, Kahlmeter G, Olsson-Liljequist B, Paterson DL, Rice LB, Stelling J, Struelens MJ, Vatopoulos A, Weber JT, Monnet DL. 2012. Multidrug-resistant, extensively drug-resistant and pandrug-resistant bacteria: an international expert proposal for interim standard definitions for acquired resistance. Clin Microbiol Infect 18:268–281. doi:10.1111/j.1469-0691.2011.03570.x 21793988

[14] Clinical Laboratory Standard Institute. 2023. Appendix C quality control strains for antimicrobial susceptibility tests page no. 290*.* M07-A13, 33^rd^ edition ed. Clinical Laboratory Standard Institute, Wayne, PA.

[15] Erica LF, Bruno GNA, Morais LL, Marin MFA, Vicente ACP. 2017. A one-step multiplex PCR to identify Klebsiella pneumoniae, Klebsiella variicola, and Klebsiella quasipneumoniae in the clinical routine. Diagn Microbiol Infect Dis 87:315–317. doi:10.1016/j.diagmicrobio.2017.01.005. 28139276

[16] Hong SS, Kim K, Huh JY, Jung B, Kang MS, Hong SG. 2012. Multiplex PCR for rapid detection of genes encoding class A carbapenemases. Ann Lab Med 32:359–361. doi:10.3343/alm.2012.32.5.359 22950072 PMC3427824

[17] Poirel L, Walsh TR, Cuvillier V, Nordmann P. 2011. Multiplex PCR for detection of acquired carbapenemase genes. Diagn Microbiol Infect Dis 70:119–123. doi:10.1016/j.diagmicrobio.2010.12.002 21398074

[18] Jayol A, Poirel L, Brink A, Villegas MV, Nordmann P. 2014. Molecular epidemiology of emerging multidrug-resistant Enterobacteriaceae. J Antimicrob Chemother 69:2861–2873. doi:10.1093/jac/dku256

[19] Banerjee T, Bhattacharjee A, Upadhyay S, Mishra S, Tiwari K, Anupurba S, Sen MR, Basu S, Kumar A. 2016. Long-term outbreak of Klebsiella pneumoniae & third generation cephalosporin use in a neonatal intensive care unit in north India. Indian J Med Res 144:622–629. doi:10.4103/0971-5916.200900 28256474 PMC5345312

[20] Koeleman JGM, Stoof J, Van Der Bijl MW, Vandenbroucke-Grauls CMJE, Savelkoul PHM. 2001. Identification of epidemic strains of Acinetobacter baumannii by integrase gene PCR . J Clin Microbiol 39:8–13. doi:10.1128/JCM.39.1.8-13.2001 11136740 PMC87671

[21] Jammeson J, Paterson D L, Sidjabat H E. 2020. The role of two-component systems in the regulation of antibiotic resistance in Gram-negative bacteria. J Antimicrob Chemother 75:251–261. doi:10.1093/jac/dkz455

[22] Borjesson S, Rebelo AR, Santos LM, Schmitt FJ, Ruiz E, Johansson A. 2018. Multiplex PCR for detection of plasmid-mediated colistin resistance determinants mcr-1, mcr-2, mcr-3, mcr-4 and mcr-5 in Enterobacteriaceae. J Antimicrob Chemother 73:2941–2948. doi:10.1093/jac/dky27.30165641

[23] Potter RF, Lainhart W, Twentyman J, Wallace MA, Wang B, Burnham CA, Rosen DA, Dantas G. 2018. Population structure, antibiotic resistance, and uropathogenicity of Klebsiella variicola. mBio 9:e02481-18. doi:10.1128/mBio.02481-18 30563902 PMC6299229

[24] Thapa A, Upreti MK, Bimali NK, Shrestha B, Sah AK, Nepal K, Dhungel B, Adhikari S, Adhikari N, Lekhak B, Rijal KR. 2022. Detection of NDM375 variants (blaNDM-1, blaNDM-2, blaNDM-3) from carbapenem-resistant Escherichia coli and Klebsiella pneumoniae: first report from Nepal. Infect Drug Resist 15. doi:10.2147/IDR.S369934 PMC9379106 35983298

[25] Maatallah M, Vading M, Kabir MH, Bakhrouf A, Kalin M, Nauclér P, Brisse S, Giske CG. 2014. Klebsiella variicola is a frequent cause of bloodstream infection in the stockholm area, and associated with higher mortality compared to K. pneumoniae. PLoS One 9:e113539. doi:10.1371/journal.pone.0113539 25426853 PMC4245126

[26] Imai K, Ishibashi N, Kodana M, Tarumoto N, Sakai J, Kawamura T, Takeuchi S, Taji Y, Ebihara Y, Ikebuchi K, Murakami T, Maeda T, Mitsutake K, Maesaki S. 2019. Clinical characteristics in blood stream infections caused by Klebsiella pneumoniae, Klebsiella variicola, and Klebsiella quasipneumoniae: a comparative study, Japan, 2014-2017. BMC Infect Dis 19:946. doi:10.1186/s12879-019-4498-x 31703559 PMC6842162

[27] Garza-Ramos U, Silva-Sánchez J, Catalán-Nájera J, Barrios H, Rodríguez-Medina N, Garza-González E, Cevallos MA, Lozano L. 2016. Draft genome sequence of a hypermucoviscous extended-spectrum-β-lactamase-producing Klebsiella quasipneumoniae subsp. similipneumoniae clinical isolate. Genome Announc 4:e00475-16. doi:10.1128/genomeA.00475-16 27389261 PMC4939778

[28] Long SW, Linson SE, Ojeda Saavedra M, Cantu C, Davis JJ, Brettin T, Olsen RJ. 2017. Whole-genome sequencing of human clinical Klebsiella pneumoniae isolates reveals misidentification and misunderstandings of Klebsiella pneumoniae, Klebsiella variicola, and Klebsiella quasipneumoniae. mSphere 2:e00290–17. doi:10.1128/mSphereDirect.00290-17 28776045 PMC5541162

[29] Mathers AJ, Crook D, Vaughan A, Barry KE, Vegesana K, Stoesser N, Parikh HI, Sebra R, Kotay S, Walker AS, Sheppard AE. 2019. Klebsiella quasipneumoniae provides a window into carbapenemase gene transfer, plasmid rearrangements, and patient interactions with the hospital environment. Antimicrob Agents Chemother 63:e02513-18. doi:10.1128/AAC.02513-18 30910889 PMC6535554

[30] Duran-Bedolla J, Garza-Ramos U, Rodríguez-Medina N, Aguilar Vera A, Barrios-Camacho H. 2021. Exploring the environmental traits and applications of Klebsiella variicola. Braz J Microbiol 52:2233–2245. doi:10.1007/s42770-021-00630-z 34626346 PMC8578232

[31] Kumar P, Takei S, Tabe Y, Miida T, Hishinuma T, Khasawneh A. 2019. Klebsiella variicola bacteremia in a patient with diabetes mellitus. J Clin Diagn Res 13. doi:10.7860/JCDR/39634.1311.

[32] Nang SC, Han M-L, Yu HH, Wang J, Torres VVL, Dai C, Velkov T, Harper M, Li J. 2019. Polymyxin resistance in Klebsiella pneumoniae: multifaceted mechanisms utilized in the presence and absence of the plasmid-encoded phosphoethanolamine transferase gene mcr-1. J Antimicrob Chemother 74:3190–3198. doi:10.1093/jac/dkz314 31365098 PMC6798846

